# INTERGENERATIONAL OCCUPATIONAL THERAPY PROGRAMMING FOR COMMUNITY-DWELLING OLDER ADULTS AND SCHOOL-AGE CHILDREN

**DOI:** 10.1093/geroni/igz038.600

**Published:** 2019-11-08

**Authors:** Robin L Chilton

**Affiliations:** Cleveland State University, Cleveland, Ohio, United States

## Abstract

Community Intergenerational Action (CIA) was a four-week pilot program designed to bring community dwelling older adults and fourth-grade students together to engage in meaningful activities within a supportive group context under the facilitation of Robin Chilton, MBA, OTR/L, and four Master of Occupational Therapy Students from Cleveland State University. A phenomenological, qualitative research design study was conducted to explore the impact and meaning of this intergenerational occupational therapy programming on the social and emotional well-being of older adults. Participant observation, journaling, and in-depth interviews were used to determine the meaning of the program to the participants. CIA was conducted using ten female elders, and fifteen children ages nine to ten years old. Each week a new theme was introduced to assist the participants in solving a mystery and included an occupation-based activity such as horticulture and crafts. The CIA program was developed in a way that would allow it to be replicated in other intergenerational settings. Students involved in this study identified helping and cooperating with the older adults throughout the program as very important to them. The students began to feel a sense of empathy and increased self-awareness after spending time with the older adults. The program allowed the older adults to reminisce about their past, and feel a sense of generativity, or contribution to the younger generation. Findings provide an opportunity for others to use similar programs to engage older adults and children in meaningful occupation that will contribute to their overall sense of social and emotional well-being.

